# Astaxanthin Vesicles Improve Alcoholic Liver Disease Through Oxidative Stress and NF‐κB Inflammatory Pathway

**DOI:** 10.1002/fsn3.72076

**Published:** 2026-07-01

**Authors:** Chenchen Li, Xintong Zhang, Diaomei Ma, Weiye Xiu, Chunran Han

**Affiliations:** ^1^ Key Laboratory of Cereal Food and Cereal Resources in Heilongjiang Province, School of Food Engineering, Harbin University of Commerce Harbin China

**Keywords:** alcoholic liver disease, astaxanthin vesicles, inflammatory, oxidative stress

## Abstract

Liver injury induced by alcoholic fatty liver disease (ALD) will eventually lead to the development of hepatocellular carcinoma. The antioxidant and anti‐inflammatory functions of astaxanthin (AST) can prevent and alleviate liver injury. In this study, AST was embedded in fatty acid vesicles to determine the effects of astaxanthin vesicles (AST‐FAV) on ALD. The results demonstrated that compared to free AST, AST‐FAV improved liver fat accumulation and oxidative damage caused by excessive drinking, and inhibited the production of pro‐inflammatory cytokines by regulating the TLRs/MyD88/NF‐κB, TNF‐α/TNFR/NF‐κB, and NLRP3/NF‐κB pathways. In summary, AST‐FAV can exert its anti‐inflammatory effect through antioxidation and multiple pathways to prevent liver injury. These findings not only highlight the potential of AST‐FAV for application in functional foods or dietary supplements but also provide a theoretical basis for further exploration of its potential as a clinical intervention strategy for the prevention or adjunctive treatment of alcoholic liver disease in humans.

## Graphical abstract

Excessive alcohol consumption can induce alcoholic liver disease (ALD), and its pathogenesis is closely associated with exacerbated hepatic oxidative stress and inflammatory responses. AST‐FAV are capable of effectively delivering AST to liver tissues, scavenging excessive reactive oxygen species, and blocking the activation of the NF‐κB signaling pathway, thereby downregulating the release of pro‐inflammatory cytokines and alleviating hepatic oxidative stress and inflammatory damage. By simultaneously modulating oxidative stress levels and the NF‐κB‐mediated inflammatory cascade, this system effectively alleviates alcohol‐induced liver injury, providing a safe and efficient novel intervention strategy for the prevention and treatment of ALD.

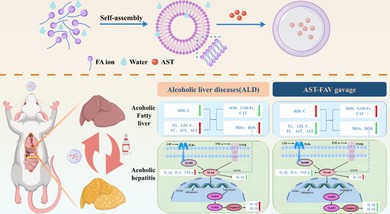

## 1 Introduction

According to a report by the World Health Organization, more than 200 million people in the world consume alcohol, accounting for 3.7% of the global population. As the main organ involved in alcohol metabolism, the liver is the most widely damaged organ. Alcoholic liver disease (ALD) occurs when ethanol intake exceeds the maximum level of complete liver metabolism (Mitra et al. 2020). ALD can progress from steatosis to alcoholic steatohepatitis (ASH) and then to liver cirrhosis and end‐stage hepatocellular carcinoma. Steatosis is a reversible pathology that can be relieved after abstinence from alcohol. ASH is characterized by infiltration of liver tissue by inflammatory cells and an increase in liver necrosis. Severe ASH is characterized by irreversible inflammation, which is one of the key nodes in the process of ALD. Liver cancer can be induced if it is not controlled at this stage. The pathogenesis of ALD includes oxidative stress, the inflammatory response, and other common pathological reactions associated with liver injury. Oxidative stress leads to the accumulation of ROS and the activation of the inflammatory signaling pathway to promote the release of inflammatory factors. The inflammatory response is a typical pathological mechanism of alcoholic liver injury, and an increase in the expression of inflammatory factors is very important in the inflammatory response (Sun et al. 2024). This inflammatory response leads to the production of the inflammasome NLRP3. The production of inflammatory cytokines is coordinated by two signals. First, lipopolysaccharide (LPS) activates the transcription factor NF‐*κ*B pathway, which in turn promotes the excessive production of inflammatory cytokines such as IL‐1*β*, IL‐6, IL‐18, and TNF‐*α* (Gao et al. 2019) and then induces lipid accumulation and apoptosis in hepatocytes. Downstream signal transduction events include MyD88‐dependent activation of NF‐*κ*B, which ameliorates alcohol‐induced liver disease. Additionally, NF‐*κ*B also activates the inflammasome. In the context of inflammation, the NLRP3‐mediated pyroptosis pathway is very important for innate immune function. The NLRP3 inflammasome is a kind of cytoplasmic polyprotein complex that can activate caspase‐1, lead to the production of the cytokines IL‐1*β* and IL‐18, and induce cell pyroptosis. ALD treatment mainly includes abstinence from alcohol, dietary therapy, and medication improvement, and in severe cases, liver transplantation is needed. ALD treatment requires the use of drugs with strong anti‐inflammatory properties (Kim 2016), but these drugs have different side effects. Therefore, alternative natural dietary supplements need to be identified to prevent and improve ALD.

Astaxanthin (AST) is a fat‐soluble ketone carotenoid that is safe to use; thus, it is classified as a “pure antioxidant” (Sandmann 2025). The AST molecule contains 2*β*‐ionone rings and 11 conjugated double bonds. It has antioxidant (Liu et al. 2020), anti‐inflammatory (Yaghooti et al. 2019), heart protection (Krestinina et al. 2020), liver protection (Yang et al. 2019), anti‐diabetic (Penislusshiyan 2020), and neuroprotective (Sharma et al. 2018) effects, and it can also prevent other diseases, making this kind of carotenoid very popular among consumers in the nutrition and healthcare market. The hepatoprotective effect of AST was confirmed in high‐fat diet‐fed mice (Jia et al. 2016) and nonalcoholic steatohepatitis (NASH) model mice (Kim et al. 2017). AST can also prevent diet‐induced obesity and liver steatosis in mice; AST is more effective than vitamin E (Ni et al. 2015). AST can inhibit the expression of oxidative stress and inflammatory factors (Han et al. 2018). AST can prevent ALD by regulating the redox balance of mitochondria (Wang et al. 2023). However, AST is vulnerable to oxidative degradation under the effect of light and temperature, resulting in the instability of its chemical structure; alterations in the chemical structure affect its biological activity and physiological function, thus limiting its practical application. Many scientists have used delivery systems to solve the problem of the instability of AST. Many delivery systems, such as liposomes (Zhao et al. 2024), emulsions (Boonlao et al. 2020), and nanoparticles (Wang et al. 2017), can improve the stability of AST. However, the greatest disadvantage of emulsions and liposomes is that they are thermodynamically unstable and prone to drug leakage, while some polymers that form nanoparticles are expensive, and the preparation process is complex. Fatty acid vesicles (FAV), which have hydrophilic and lipophilic properties, are closed lipid bilayer colloidal suspensions formed from a salt solution of fatty acids through self‐composition (Kaur et al. 2023). Compared to liposomes and emulsions, fatty acid vesicle membranes exhibit higher fluidity and greater molecular packing freedom. This allows AST to more readily insert and embed into the hydrophobic regions of the bilayer during the loading process, thereby enhancing encapsulation efficiency. Additionally, the amphiphilic nature of fatty acids confers good affinity for hydrophobic AST, promoting its stable encapsulation within the bilayer membrane (Jang et al. 2023). Furthermore, fatty acid molecules possess a relatively simple chemical structure without the ester bonds susceptible to hydrolysis, rendering them less prone to hydrolysis compared to liposomes. This characteristic provides better protection for AST, preventing its degradation (Nowak et al. 2023). FAV can also be used as an anti‐inflammatory carrier for delivering anti‐inflammatory drugs (Salama and Aburahma 2015). Therefore, FAV can be used as a substitute for liposomes as a carrier for the oral administration of drugs that cannot be absorbed easily (Zahariev et al. 2023) and achieve a sustained release effect (Zakir et al. 2010).

In this study, mice with ALD were administered AST‐FAV. The expression levels of liver lipid accumulation and oxidative stress‐related indicators were evaluated by enzyme‐linked immunosorbent assay (ELISA). The levels of inflammation‐related proteins were determined by Western blotting (WB) and immunofluorescence (IF) assays. Finally, the mechanism underlying the action of AST‐FAV on ALD was discussed.

## 2 Materials and Methods

### 2.1 Materials and Reagents

Male Kunming (KM) mice (SPF class) (6 weeks old; weight: 18–24 g) were used by Changchun, China (animal license number: SCXK [ji]‐2020Muth0002; quality certificate No. 202000034164) to conduct experiments. AST (purity > 96%) was purchased from Shaanxi Jinkangtai Biotechnology Co. Ltd.; arachidonic acid (purity 40%) was purchased from Shanghai McLean Biochemical Technology Co. Ltd. Span 60, Tween 85 (analytical purity) were purchased from Tianjin Fuyu Fine Chemical Co. Ltd.; anhydrous ethanol and chloroform (analytical purity) were purchased from Tianjin Tianli Chemical Reagent Co. Ltd. Alanine transaminase (ALT), aspartate transaminase (AST), triglyceride (TG), total cholesterol (TC), malondialdehyde (MDA), high‐density lipoprotein cholesterol (HDL‐C), low‐density lipoprotein cholesterol (LDL‐C), catalase (CAT), superoxide dismutase (SOD), reactive oxygen species (ROS), and glutathione peroxidase (GSH‐Px) detection kits were purchased from Nanjing Jiancheng Institute of Biological Engineering Detection kits for interleukin‐1*β* (IL‐1*β*), interleukin‐6 (IL‐6), interleukin‐10 (IL‐10), interleukin‐18 (IL‐18), NF‐*κ*B, and TNF‐*α* were purchased from Jingmei Biotechnology Co. Ltd. The high‐fat diet (45% kcal from fat, catalog number 1145DM) was purchased from Beijing Boaigang Biotechnology Co. Ltd.

### 2.2 Instruments and Equipment

WH‐2 vortex mixer: Shanghai Luxi Analytical Instrument Factory Co. Ltd. WD‐9405 decolorizing shaker: Beijing June 1st Instrument Factory. ZLI‐9305 histochemical pen: Zhongshan Jinqiao DYY‐6C electrophoresis instrument, DYCZ‐400D transfer electrophoresis slot, DYCZ‐24DN vertical electrophoresis slot: Beijing June 1st Instrument Factory. Cmax plus enzyme labeling instrument: Molecular Company; TGL‐16c desktop centrifuge: Shanghai Anting Scientific Instrument Factory; HSC‐2015L frozen centrifuge: Xinzhi; IMS‐20 Ice maker: Changshu Xueke Electric Appliance Co. Ltd.; HS‐25 shift shaker, DTH‐100 dry thermostat: Hangzhou Miou Instrument Co. Ltd.; AX‐II cassette: Guangdong Yuehua Medical Equipment Factory; 9000F MarkII scanner: Canon.

### 2.3 Method

#### 2.3.1 Preparation of AST‐FAV

AST‐FAV was prepared following the method described by Yadav et al. (2025) with slight modifications. The organic solvent (chloroform: ethanol = 3:2), surfactant, arachidonic acid, and AST were added to the round‐bottom flask, which was shaken until the AST was completely dissolved in the organic solvent. The round‐bottom flask was placed on a rotary evaporation meter, a water bath was used at 30°C, and the organic dissolving agent was removed under reduced pressure until an orange‐red film formed at the bottom of the round‐bottom flask. To prevent the residue of organic solvents (chloroform and ethanol), the round‐bottom flask was placed in a vacuum drying box at 25°C overnight. After overnight treatment, 20 mL of the prepared 0.1 mol/L phosphate buffer solution was added to the round‐bottom flask, and the mixture was treated for 20 min in a 40 W ultrasonic cleaner at 25°C. All films at the bottom of the bottle were rehydrated to form AST‐FAV. After hydration, the formed AST‐FAV membrane was filtered through a 0.45‐μm filter.

The appearance and structural characterization of AST‐FAV are shown in Figure S1A–D. Through single‐factor experiments and response surface methodology optimization, the optimal preparation conditions for AST‐FAV were determined as follows: astaxanthin concentration of 0.14 mg/mL, surfactant‐to‐fatty acid ratio of 1:1, hydrophilic–lipophilic balance (HLB) value of 7, phosphate buffer solution pH of 7.0, and hydration time of 21 min. Under these optimal conditions, the encapsulation efficiency (EE) of AST‐FAV reached 89.58% ± 1.47%, with a drug loading capacity (DL) of 21.08%.

Transmission electron microscopy and particle size analysis revealed that AST‐FAV had a good spherical structure, with an average particle size of 131.74 ± 2.74 mV, a PDI of 0.25 ± 0.01, and a zeta potential of −38.52 ± 2.31 mV. The particle size structure and red color are shown in Figure S1C,D. A standard curve for AST was established via high‐performance liquid chromatography, and the levels of AST in the AST‐FAV were measured via demulsification. The in vitro oral, gastric, and intestinal digestion retention rates of AST‐FAV and Oil AST were 74.99% ± 4.37% and 44.19% ± 2.21%, respectively.

#### 2.3.2 Animal Feeding and Model Establishment

The experimental mice were raised in a specific pathogen‐free (SPF) environment with constant temperature and humidity. The animal experimental procedures followed the animal ethics standards and were approved by the Animal Ethics Committee of Harbin University of Commerce (Approval No. HSDU2024065, Harbin, China). Water and food were provided normally, and adaptive feeding was performed for 1 week before the experiment was conducted. The feeding environment was as follows: the room temperature was controlled at 25°C–27°C, the humidity was maintained at 50%–60%, the lighting conditions followed a 12/12‐h light/dark cycle, and the cushion was changed twice a week.

The experimental method was adapted from the study by Gao et al. (2022) with appropriate modifications to meet the specific needs of this study. A total of 80 mice were randomly divided into eight groups: normal control (NC), model control (MC), positive control (PC, 20 mg/kg silymarin), FAV group (100 mg/kg FAV), and free astaxanthin control group (AST, 20 mg/kg). In addition, 50 mg AST‐FAV (containing 10 mg AST), 75 mg AST‐FAV (containing 15 mg AST), and 100 mg AST‐FAV (containing 20 mg AST) were designated as the low‐dose (AST‐FAV[L]), medium‐dose (AST‐FAV[M]), and high‐dose (AST‐FAV[H]) groups, respectively. Each group consisted of 10 mice, and the total experimental period was 9 weeks (Liang et al. 2018).

**FIGURE 1:**
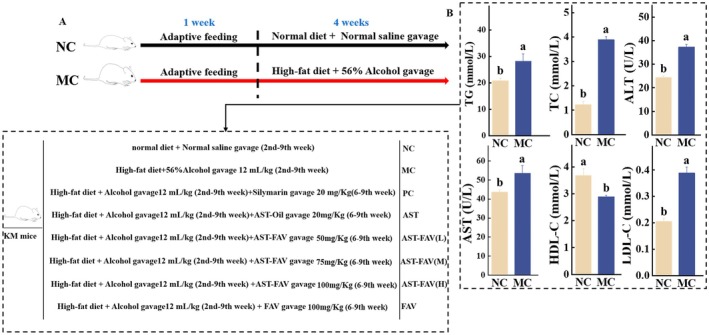
Establishment of the ALD mouse model. (A) Schematic of the experimental design and group assignment. (B) Serum lipid and liver function parameters in normal control (NC) and model control (MC) groups.

The alcoholic fatty liver model was established according to the method described by Li et al. (2021), with a total treatment period of 9 weeks. The first week was designated as the acclimation period. Subsequently, a four‐week induction phase was carried out. During this phase, except for the NC group, which received normal chow and drinking water, all other groups were fed a high‐fat diet (45% kcal from fat, 35% kcal from carbohydrates, and 20% kcal from protein) and administered 56% (*v*/*v*) alcohol daily via gavage to establish the alcoholic fatty liver mouse model. After 5 weeks of modeling, three mice were randomly selected from each group, anesthetized by intraperitoneal injection of Avertin (15 μL/g body weight), and blood samples were collected from the orbital sinus. As shown in Figure 1B, the results indicated that the levels of triglycerides (TG), total cholesterol (TC), alanine aminotransferase (ALT), aspartate aminotransferase (AST), and low‐density lipoprotein cholesterol (LDL‐C) in the blood were significantly elevated. Thereafter, a 4‐week treatment phase was initiated, during which gavage administration was performed in addition to continued high‐fat feeding. Body weight changes were recorded every 2 days, and all mice were fasted for 12 h (with free access to water) before the end of the experiment. After 9 weeks, all mice were again anesthetized by intraperitoneal injection of Avertin (15 μL/g body weight). Blood samples were collected from the orbital sinus, and the mice were then euthanized. A portion of the liver tissue was fixed in 4% paraformaldehyde, and the remaining tissue was immediately frozen in liquid nitrogen and stored at −80°C for subsequent analysis.

#### 2.3.3 Weight and Liver Index

During the experiment, the weights of the mice were recorded every other day before gavage. After the mice were killed, the liver was weighed, and the liver index was calculated.

$$\text{Liver index}=\frac{\text{Liver weight}\;\left(g\right)}{\text{Body weight}\;\left(g\right)}\times 100\%$$

#### 2.3.4 Determination of Serum Indices

Blood samples were collected from the retro‐orbital venous plexus of each mouse, allowed to stand for 30 min, and then centrifuged at 1500 r/min for 15 min at 4°C to obtain serum. Serum levels of HDL‐C, LDL‐C, TC, TG, ALT, and AST were determined using commercial assay kits from Nanjing Jiancheng Bioengineering Institute (Nanjing, China). All procedures were operated according to the manufacturer's instructions of the corresponding commercial assay kits.

#### 2.3.5 Determination of Oxidative Stress Indices in Liver Tissue

Liver tissue (0.2 g) was excised, rinsed with ice‐cold phosphate‐buffered saline (PBS; 0.01 M, pH 7.4) to remove residual blood, and blotted dry with filter paper. Pre‐chilled PBS was added at a tissue weight‐to‐buffer volume ratio of 1:9 (*w*/*v*), and the mixture was homogenized on ice using a tissue homogenizer at 10,000 r/min for 30 s, followed by a 30 s pause, repeated three times, to yield a 10% (*w*/*v*) tissue homogenate. The homogenate was centrifuged at 3000 r/min for 15 min at 4°C, and the resulting supernatant was collected for subsequent analyses (Wang et al. 2024). The levels of reactive oxygen species (ROS) and malondialdehyde (MDA), as well as the activities of catalase (CAT), superoxide dismutase (SOD), and glutathione peroxidase (GSH‐Px), in the supernatant were determined according to the manufacturer's instructions for the respective commercial assay kits (Nanjing Jiancheng Bioengineering Institute, Nanjing, China).

#### 2.3.6 Detection of Lipopolysaccharide (LPS) and Related Inflammatory Factors via ELISA

The experiment was conducted following the instructions provided with the ELISA kit, and the levels of LPS, IL‐1*β*, IL6, IL‐10, IL‐18, NF‐*κ*B, and TNF‐*α* in the serum of the mice were calculated.

#### 2.3.7 Pathological Observation of the Liver

Liver tissues were processed for paraffin embedding and sliced, after which routine hematoxylin–eosin (HE) staining was performed, and the samples were observed under a light microscope.

#### 2.3.8 Oil Red O (ORO) Staining of the Liver

The fixed liver tissue was embedded with a frozen section embedding agent. After the sections were frozen, the liver tissue was stained with ORO and observed under a light microscope.

#### 2.3.9 Protein Detection by Western Blotting (WB)

Total proteins were extracted from the cryopreserved liver tissues via ultrasonic treatment using RIPA lysis buffer. Following the determination of total protein concentration, the sample volume was adjusted to ensure a consistent loading amount across all groups. After the protein was denatured at a high temperature (70°C), a 10% separation gel was prepared for sodium dodecyl sulfate‐polyacrylamide gel electrophoresis (voltage 80 V, electrophoresis time 20 min, voltage 120 V, and electrophoresis time 40 min) and then transferred to an NC membrane at 90 V for 90 min. After separation for 90 min with 5% skim milk powder, TNFR, MyD88, TLR2, TNF‐*α*, and *β*‐actin were added, and the mixture was incubated overnight at 4°C. After the membrane was washed, the corresponding secondary antibodies were added and incubated at room temperature for 1 h. For enhanced chemiluminescence (ECL) development, a Chemi Doc XRS gel imaging system was used for signal scanning, and the ImageJ software was used for analyzing the images to obtain the gray value of the protein band. The expression level of the target protein was expressed as the ratio of the gray value of the target protein to that of the internal reference protein (β‐actin).

#### 2.3.10 Immunofluorescence (IF) Staining

The fixed liver tissue was prepared for dewaxing and antigen repair. The samples were blocked with BSA for 45 min at room temperature, and then the diluted primary antibodies were added and incubated overnight at 4°C. The secondary fluorescent antibodies were added the following day, and the samples were incubated in the dark for 2 h. Then the samples were stained with DAPI and sealed. The expression of NLRP3 and Caspase‐1 proteins was observed via fluorescence microscopy.

### 2.4 Statistical Analysis

All experimental data are presented as mean ± standard deviation (SD). Comparisons among multiple groups were performed using one‐way analysis of variance (ANOVA), followed by Tukey's honest significant difference (HSD) test for post hoc multiple comparisons. All statistical analyses were conducted using SPSS software (IBM Corp., Armonk, NY, USA). A *p* < 0.05 was considered statistically significant. Graphs were prepared using Origin 2021 software (OriginLab Corp., Northampton, MA, USA).

## 3 Results

### 3.1 Effects of AST‐FAV on Alcohol‐Induced Liver Injury

#### 3.1.1 Effects of AST‐FAV on the Liver Indices of the Mice

**FIGURE 2:**
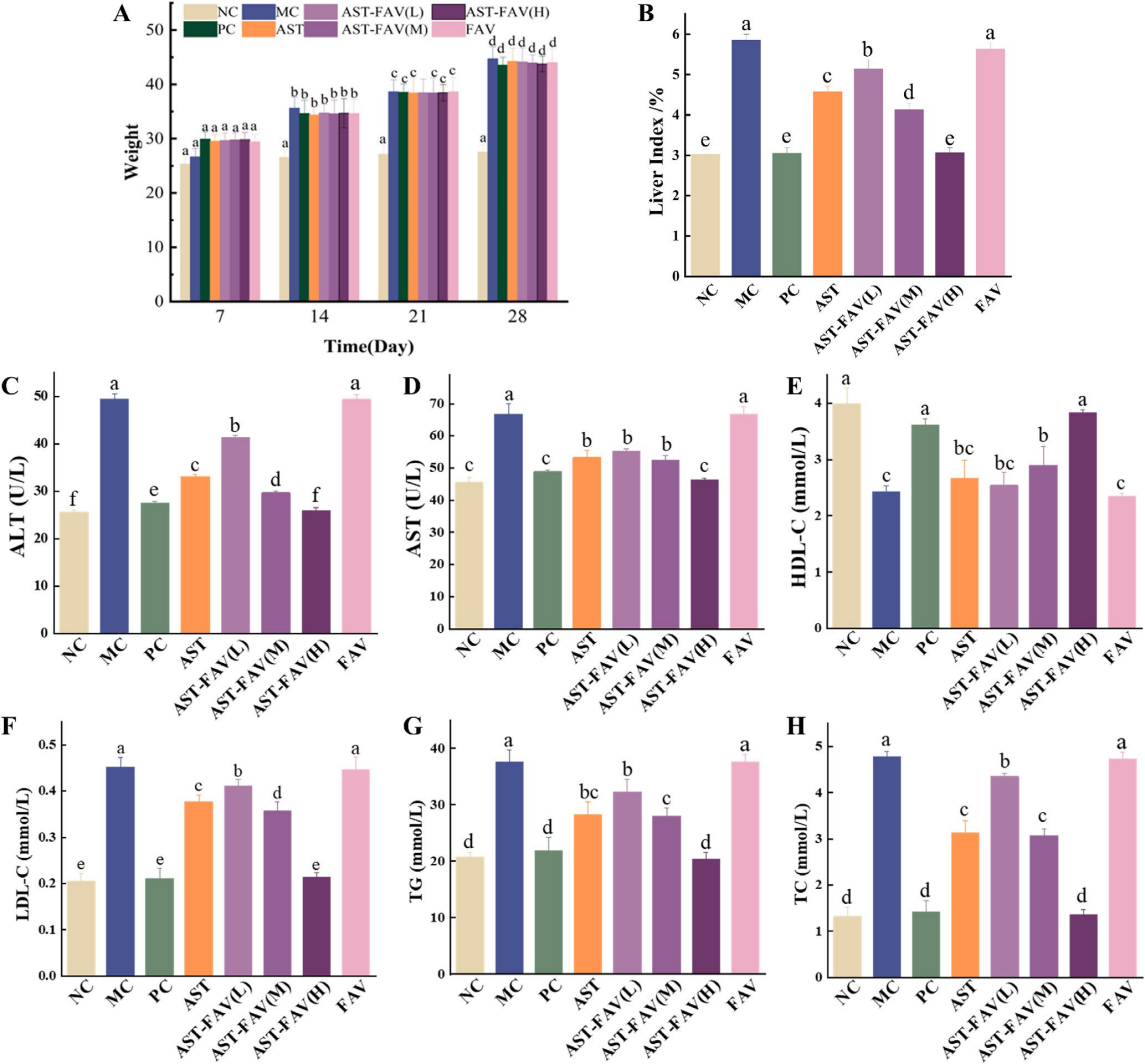
Effects of AST‐FAV on the weight (A), liver index (B), ALT (C), AST (D), HDL‐C (E), LDL‐C (F), TG (G), and TC (H) of ALD mice. The data are expressed as the means ± SDs (*n* = 8). Significant differences (*p* < 0.05) are indicated with different letters, and the letter order indicates a decreasing average.

As shown in Figure 2B, the liver index (liver weight/body weight × 100%) was significantly higher in the MC group than in the NC group (*p* < 0.05), indicating that alcohol combined with a high‐fat diet induced liver swelling in mice and confirming successful model establishment. Compared with the MC group, both the free AST group and the AST‐FAV group showed a significant reduction in liver index (*p* < 0.05), demonstrating that both free AST and AST‐FAV effectively alleviated the elevation of liver index caused by alcohol and high‐fat diet. Furthermore, AST‐FAV exerted a significantly better improvement effect on liver index than free AST (*p* < 0.05).

#### 3.1.2 Effects of AST‐FAV on the Serum Biochemical Indices of ALD Mice

The enzymes ALT and AST are found mainly in hepatocytes, and elevated blood levels usually indicate liver injury or inflammation. As shown in Figure 2C,D, under normal physiological conditions, the levels of ALT and AST in the serum were lower. When the liver was stimulated by alcohol and a high‐fat diet, the levels of ALT and AST in the serum increased significantly (*p* < 0.05), indicating that alcohol plus a high‐fat diet led to liver damage in the mice. At the same dose, the levels of ALT and AST in the serum of the AST‐FAV group were 7.16 and 6.90 U/L lower than those in the free AST group, respectively, indicating that AST‐FAV can more effectively improve liver injury caused by alcohol and a high‐fat diet.

Additionally, ALD can affect fat metabolism. HDL‐C, LDL‐C, TG, and TC in serum are important indicators of fat metabolism, and these indicators are altered after severe liver damage. As shown in Figure 2E–H, compared to those in the NC group, the levels of LDL‐C, TG, and TC in the serum of the mice in the MC group were significantly greater, whereas the levels of HDL‐C was significantly lower (*p* < 0.05). These results match the blood lipid characteristics of ALD. Compared to the MC group, all treatment groups significantly decreased LDL‐C, TG, and TC levels, while HDL‐C levels increased significantly (*p* < 0.05), especially in the AST‐FAV (H) group, where the indicators were not significantly different from those in the NC group (*p* > 0.05), and were significantly higher than those in the free astaxanthin group (*p* < 0.05). These results indicated that AST‐FAV can reduce blood lipid levels in ALD mice, alleviate lipid metabolism disorders, and reduce the risk of cardiovascular disease in ALD patients.

#### 3.1.3 Effects of AST‐FAV on the Liver Histomorphology of ALD Model Mice Induced by Alcohol and Diet

**FIGURE 3:**
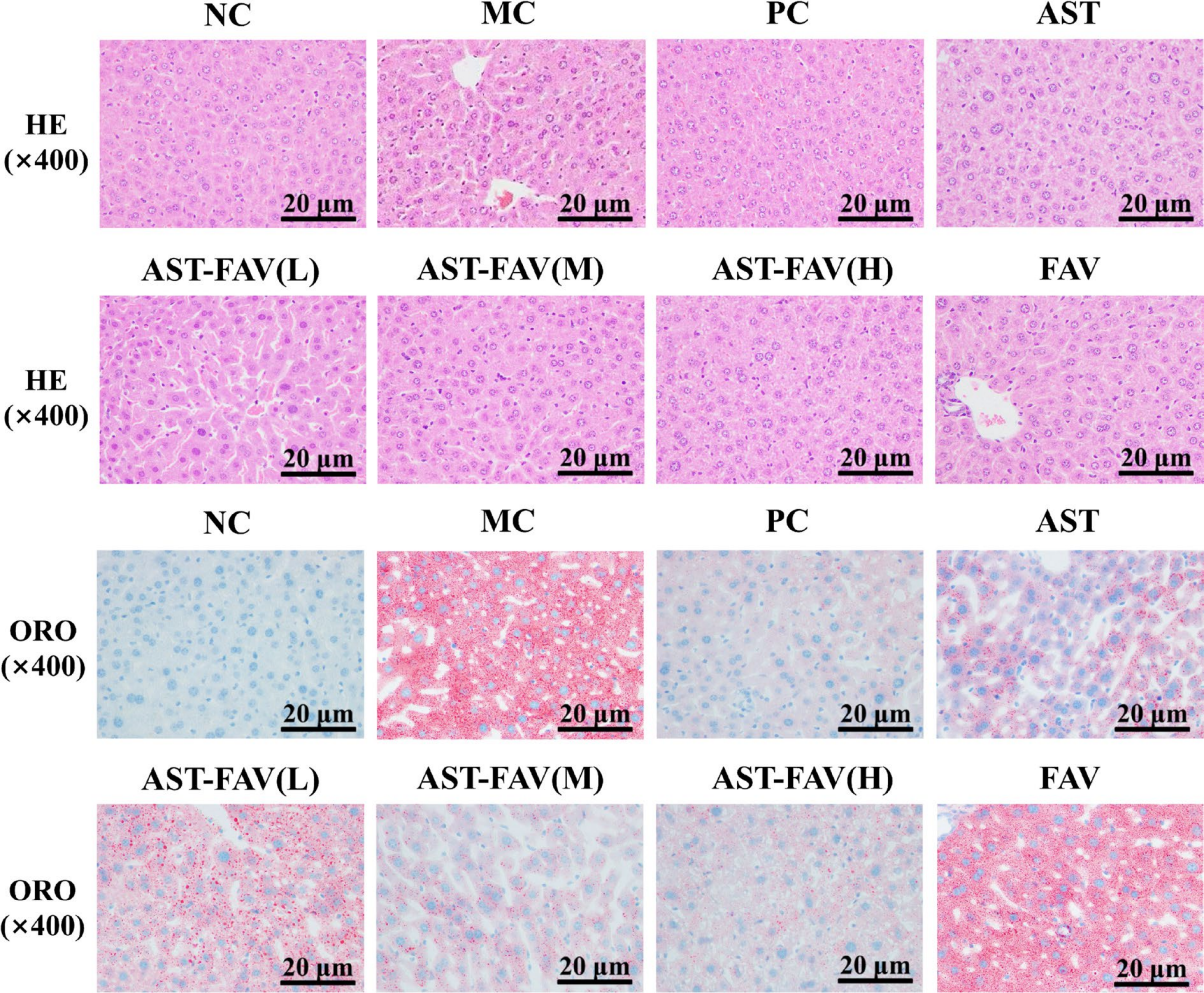
Histological analysis of livers from mice with fatty liver disease (×400) using HE and ORO staining.

The effects of AST‐FAV on the liver histomorphology of ALD mice are shown in Figure 3. HE staining revealed that the hepatic lobules in the NC group were regular and intact, with an orderly arrangement of cells and no inflammatory cell infiltration. Fat vacuoles of different sizes appeared in the MC group, and lipid accumulation around the nucleus increased significantly. Compared to the MC group, the AST‐FAV administration group presented a significant reduction in the number and size of fat vacuoles. As the dose of AST‐FAV increased, the number of cytoplasmic fat vacuoles decreased. The liver tissue morphology of the AST‐FAV (H)‐treated group was similar to that of the NC group. ORO staining is an important tool for evaluating ALD and other diseases, in which lipid accumulation in hepatocytes is a significant feature. The ORO staining results revealed that, compared to those in the NC group, the livers of the mice in the MC group showed a prominent accumulation of red lipid droplets, while the liver nuclei of the mice in the NC group were blue, and no prominent red lipid droplets were found in the liver cells. Compared to the MC group, the AST‐FAV group presented a decrease in the area stained for liver lipid deposition, which was linearly related to the dosage and tended toward the PC group. To summarize, these findings suggested that AST‐FAV can alleviate alcohol‐induced liver damage.

### 3.2 Effects of AST‐FAV on Oxidative Stress in the Livers of ALD Mice

**FIGURE 4:**
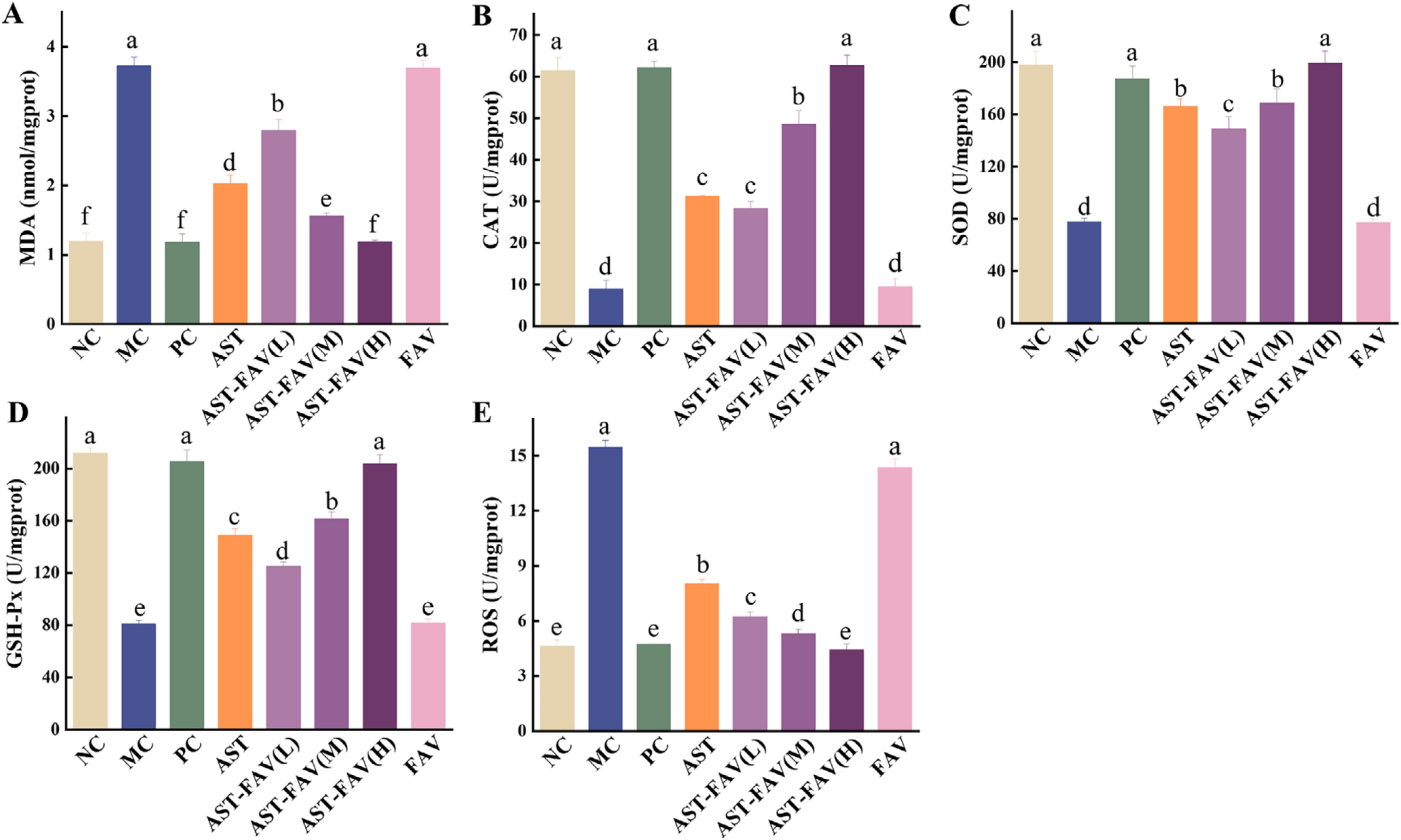
Effect of the AST‐FAV on MDA (A), CAT (B), SOD (C), GSH‐Px (D), and ROS (E) in mice with ALD. The data are expressed as the means ± SDs (*n* = 8). Significant differences (*p* < 0.05) are indicated with different letters, and the letter order indicates a decreasing average.

Alcohol produces excessive ROS and free radicals and consumes a large amount of endogenous antioxidants to induce oxidative stress through the liver metabolic pathway, which is an important factor leading to ALD. As shown in Figure 4A–E, compared to the MC group, the free AST group presented lower MDA and ROS activities and higher SOD, GSH‐Px, and CAT activities to reduce oxidative stress, indicating that AST effectively inhibited oxidative stress and liver lipid peroxidation and promoted liver protection and antioxidation. Compared to the free AST group, the AST‐FAV group presented a significant increase in CAT activities (*p* < 0.05), a significant increase in SOD and GSH‐P activity (*p* < 0.05), and a significant decrease in MDA and ROS levels (*p* < 0.05), indicating that AST‐FAV can more effectively inhibit liver oxidation caused by alcohol intake in mice. By improving liver antioxidant capacity and inhibiting the degree of liver oxidative damage, AST‐FAV can alleviate liver inflammation. Briefly, the results showed that AST‐FAV can maintain the cell redox balance by regulating SOD and CAT, neutralize harmful ROS, exhibit high antioxidant activity, and effectively reduce the level of oxidative stress in vivo.

### 3.3 Effects of AST‐FAV on Inflammatory Cytokines and Protein Expression in ALD Mice

#### 3.3.1 Effects of AST‐FAV on the Levels of Serum Inflammatory Factors and Lipopolysaccharides in ALD Mice

**FIGURE 5:**
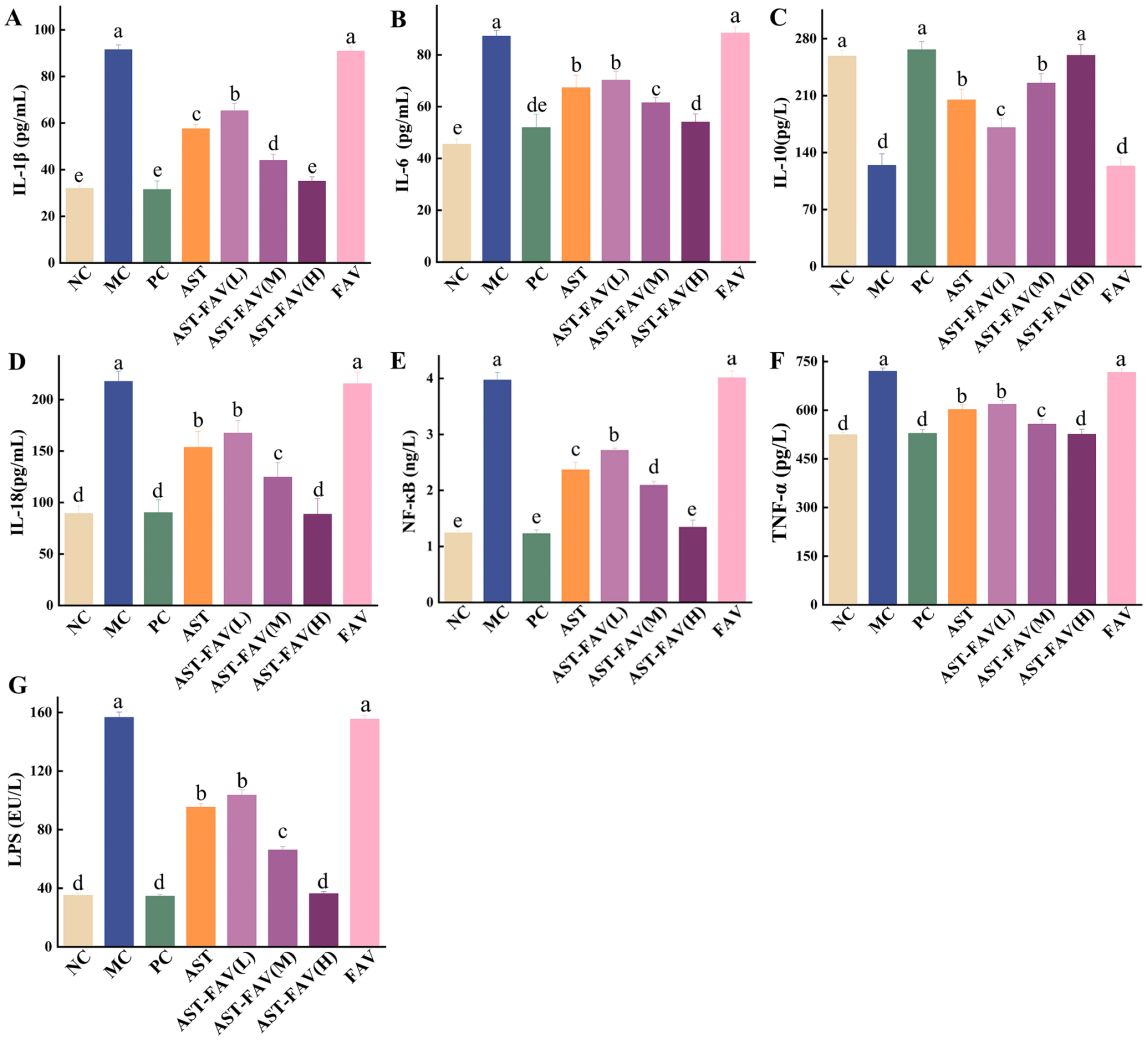
Effects of AST‐FAV on serum levels of inflammatory factors and lipopolysaccharide in ALD mice: IL‐1β (A), IL‐6 (B), IL‐10 (C), IL‐18 (D), NF‐κB (E), TNF‐α (F), and LPS (G). The data are expressed as the means ± SDs (*n* = 8). Significant differences (*p* < 0.05) are indicated with different letters, and the letter order indicates a decreasing average.

Alcohol stimulates the secretion of inflammatory factors, including various interleukins (IL‐1*β*, IL‐6, IL‐10, and IL‐18), NF‐*κ*B, TNF‐*α*, and LSP, which play important roles in the pathogenesis of ALD. As shown in Figure 5A–F, the expression and LSP levels of the proinflammatory cytokines IL‐1*β*, IL‐6, IL‐18, TNF‐*α*, NF‐*κ*B, and LSP in the MC group were significantly greater than those in the NC group (*p* < 0.05), whereas the expression of the anti‐inflammatory factor IL‐10 was significantly lower (*p* < 0.05). Compared to the same dose of free AST, AST‐FAV significantly decreased the levels of proinflammatory factors and LSP and significantly increased the expression of anti‐inflammatory factors (*p* < 0.05), indicating that AST‐FAV may have better anti‐inflammatory properties in ALD. Additionally, a dose‐dependent relationship was found. A higher concentration of AST‐FAV was associated with a greater anti‐inflammatory effect, and the anti‐inflammatory effect of AST‐FAV‐H was significantly superior to that of the free astaxanthin group (*p* < 0.05). In conclusion, the mechanism underlying the protective effect of AST‐FAV in ALD mice may be related to the regulation of the balance of inflammatory mediators and inflammatory factors.

#### 3.3.2 Effects of AST‐FAV on the Expression of Inflammation‐Related Proteins in the Liver Tissue of ALD Mice

**FIGURE 6:**
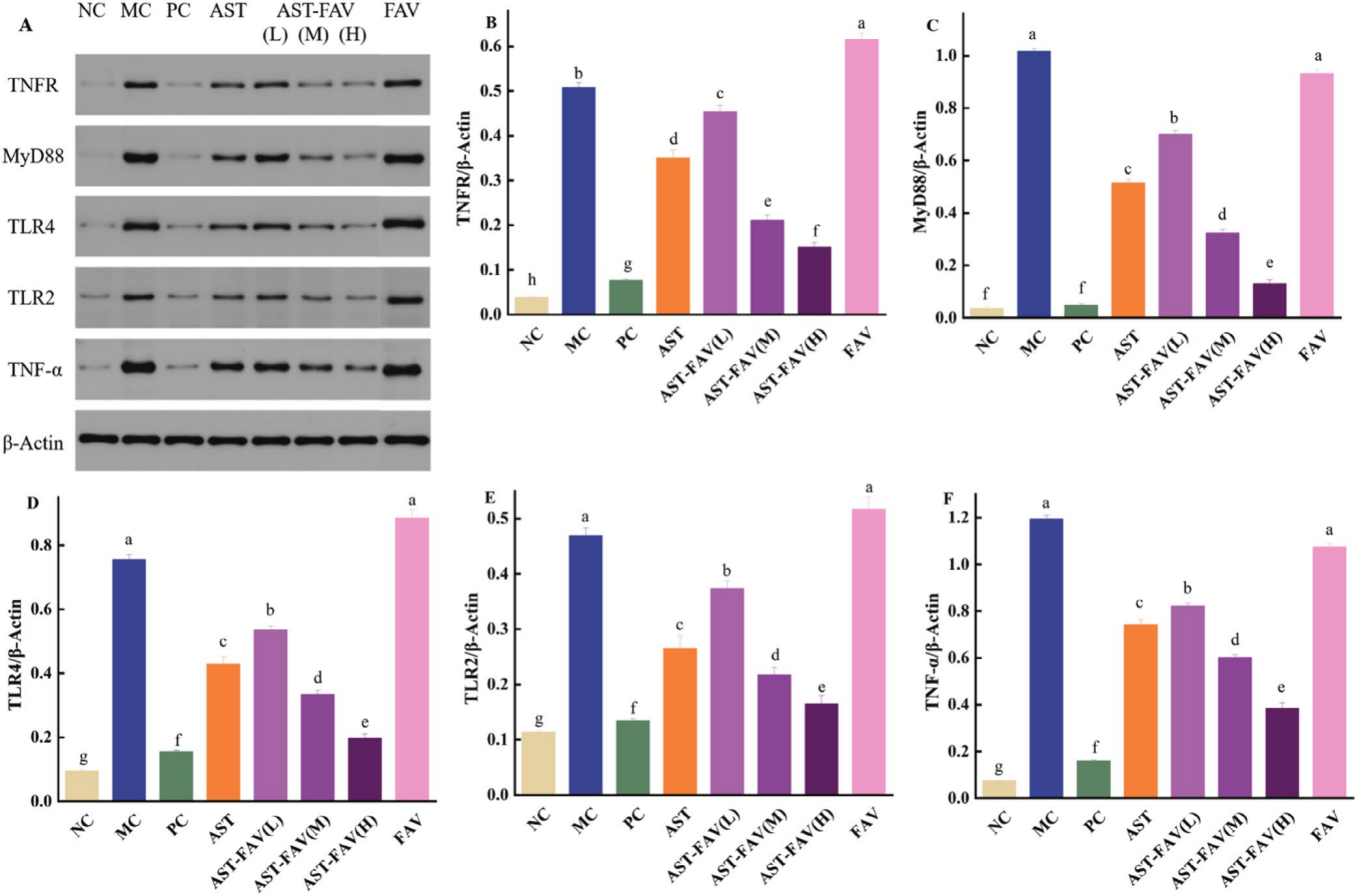
The effect of AST‐FAV on the expression of inflammation‐related proteins in the liver tissue of ALD mice: Western blot analysis (A) and relative expression levels of TNFR (B), MyD88 (C), TLR4 (D), TLR2 (E), and TNF‐α (F). The data are expressed as the means ± SDs (*n* = 8). Significant differences (*p* < 0.05) are indicated with different letters, and the letter order indicates a decreasing average.

To understand the mechanism involved in the improvement of inflammation in ALD model mice by AST‐FAV, inflammatory markers, including TNFR, MyD88, TLR4, TLR2, and TNF‐*α*, were detected by Western blotting. The results are shown in Figure 6A–F. Compared to those in the NC group, the protein expression levels of TNFR, MyD88, TLR4, TLR2, and TNF‐α in the liver tissue of the MC group were significantly greater (*p* < 0.05), which indicated that there was an inflammation‐related cascade in the MC group. The expression levels of TNFR, MyD88, TLR4, TLR2, and TNF‐*α* in the liver tissue of the mice in the free AST group were lower than those in the MC group (*p* < 0.05), indicating that AST may delay the progression of the disease by downregulating the release of inflammatory factors in the liver tissue of ALD model mice. At the same dose, the expression levels of the inflammation‐related proteins TNFR, MyD88, TLR4, TLR2, and TNF‐α in the liver tissue of the mice in the AST‐FAV group were significantly lower than those in the free AST group. These findings indicated that the AST‐FAV group presented stronger inflammatory inhibition.

#### 3.3.3 Effect of AST‐FAV on the Expression of NLRP3 and Caspase‐1 in the Liver Tissue of Mice With ALD

**FIGURE 7:**
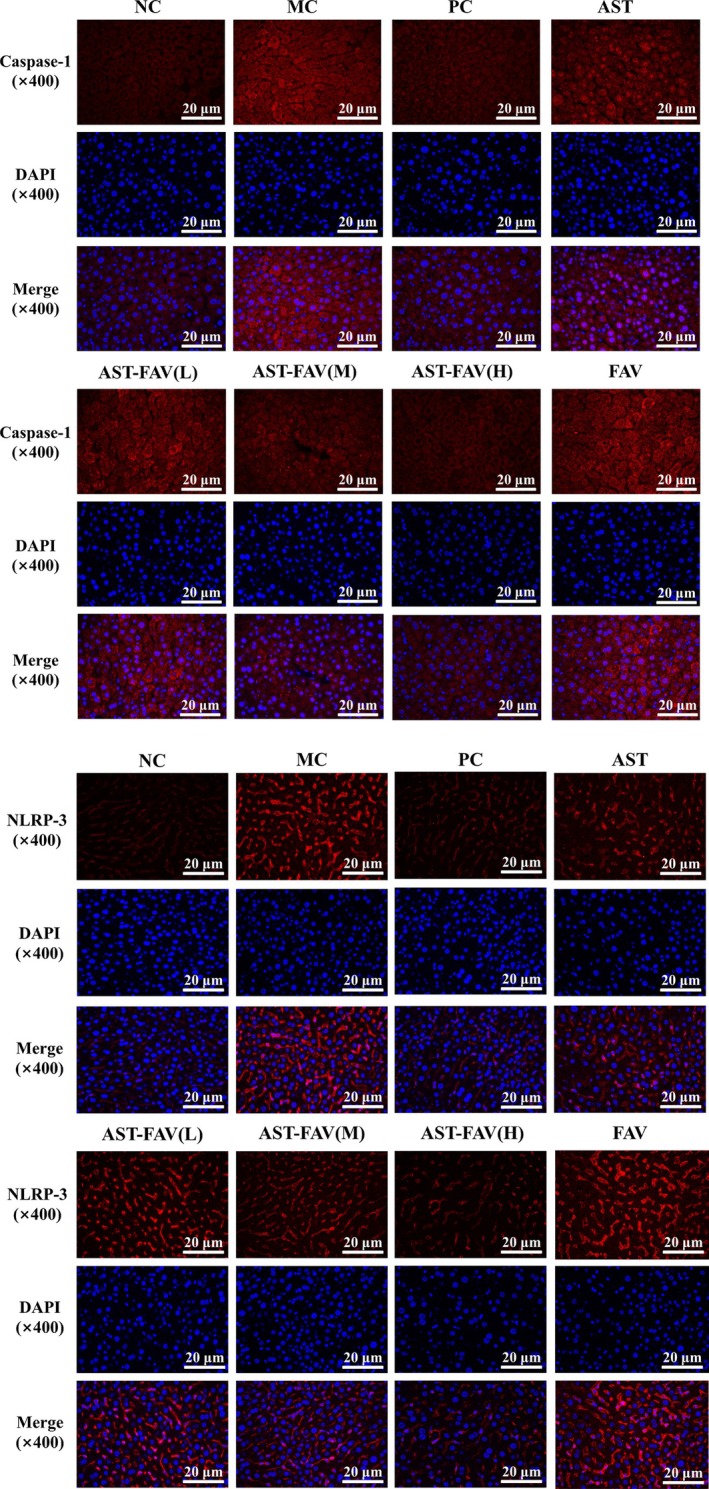
Effect of AST‐FAV on the expression levels of NLRP3 and caspase‐1 in the liver tissues of mice with ALD (400×).

An increase in the inflammatory response is usually marked by increased expression of key factors in the inflammatory pathway. As a highly representative dependent cell death pathway in the family, caspase‐1 can be activated by the classic NLRP3 inflammasome. As shown in Figure 7, the fluorescence intensity of NLRP3 and caspase‐1 in the area near the central vein of the liver in the MC group was significantly greater than that in the NC group (*p* < 0.05), indicating that the ALD mouse model induced the assembly and activation of the NLRP3 inflammasome and induced the caspase‐1 pathway in classic cells. Compared to the MC group, the free AST group and the AST‐FAV group showed good inhibitory effects on the levels of NLRP3 and caspase‐1, especially the AST‐FAV (H) group (*p* < 0.05). The results showed that AST‐FAV significantly inhibited NLRP3 and caspase‐1 in the livers of ALD model mice and improved ALD by inhibiting the activation of inflammasomes in the liver. These findings collectively demonstrate that AST‐FAV consistently outperforms free AST in terms of biochemical, histological, and molecular markers.

## 4 Discussion

ALD is a global liver disease problem. The progression of ALD can be related to oxidative stress markers, the inflammatory cascade, and a considerable increase in the inflammasome levels (Wu et al. 2019). Steatosis is the initial stage of ALD, and inflammation is the main factor promoting the development of ALD (Braillon and Naudet 2022). The treatment of ALD is a major challenge worldwide. The mechanism of ALD is not completely clear but involves various complex mechanisms. Mild ALD patients mainly recover through abstinence from alcohol and nutritional therapy, while moderate ALD patients mainly undergo drug intervention therapy, but the effect is partly satisfactory and is accompanied by multiple side effects. Therefore, natural products with antioxidant and anti‐inflammatory properties have attracted considerable research interest. Natural products rich in ketocarotenoids help slow the development of ALD (Clugston 2020). AST, a typical keto carotenoid, is abundant in many marine organisms and has antioxidant and anti‐inflammatory effects (Shen et al. 2014). It has hepatoprotective effects in various liver injury models (Wu et al. 2019; Zhou et al. 2022). However, AST has poor water solubility and stability, and most researchers encapsulate it in liposomes, emulsions, and nanoparticles to solve the problems of water solubility and stability. However, the process of encapsulating AST with these carriers is complex and easy to leak. FAV can be used as carriers of anti‐inflammatory drugs and has sustained release effects. In this study, we showed that AST encapsulated in FAV can more effectively alleviate ALD caused by oxidative stress and inflammation. Previous studies have shown that ALD leads to excessive deposition of TG, while a decrease in HDL‐C synthesis and secretion promotes an increase in LDL‐C synthesis, leading to an increase in TC levels, a decrease in TG liver transport, lipid metabolism disorders, and an increase in liver fat production (Zuo et al. 2019). AST and ALT are important enzyme indices of liver function. In the case of hepatocyte injury, these serum enzymes can be released into the serum, increasing their serum levels (Chen, Jiang, et al. 2021). In this study, compared to the serum levels of ALT, AST, LDL‐C, TG, TC, and HDL‐C in the free AST group, the serum levels of ALT, AST, LDL‐C, TG, and TC in the AST‐FAV group were significantly lower than those in the free AST‐FAV group, whereas the level of HDL‐C was significantly higher in the AST‐FAV group (*p* < 0.05). The results showed that AST‐FAV had the strongest protective effect on alcoholic liver injury at the same dose. Through histomorphological examination of the liver, ALD led to lipid accumulation in the liver, which was characterized by an increase in the number of hepatic fat droplets and hepatocyte enlargement, whereas AST‐FAV reduced hepatic steatosis in ALD mice. These findings suggested that AST‐FAV has a hepatoprotective effect and reduces fat infiltration in hepatocytes, which is related to the regulation of serum enzymes and reduction in liver size in ALD mice. This finding matched the results of another study (Wu et al. 2019).

Lipid metabolism disorders and lipid deposition can lead to high activities of oxidative stress in liver tissue and promote inflammatory reactions (Yang et al. 2025). Oxidative stress damage produces a large amount of ROS, causes lipid peroxidation of the liver cell membrane to accumulate a large amount of MDA, rapidly consumes the endogenous antioxidants SOD, GSH‐Px, and CAT, and exacerbates liver oxidative damage (Buko et al. 2021). In this study, oxidative stress was evaluated with the levels of ROS, MDA, and the activities of SOD, GSH‐Px, and CAT in the livers of ALD mice as the main promoters of ALD. Compared to free AST, AST‐FAV had greater SOD and GSH‐Px enzyme activities and CAT activities, whereas the ROS and MDA levels decreased significantly (*p* < 0.05). The protective effect of AST‐FAV on redox reactions is attributed to its ability to increase the level of endogenous antioxidants, reduce the level of MDA in hepatocytes, scavenge excessive ROS, and protect the liver from alcohol‐induced oxidative damage. This finding is similar to that reported in other studies on the protective effect of AST nanoparticles on ALD (Lv et al. 2025).

A complex relationship exists between the inflammatory response and oxidative stress, and they promote the development of each other. The inflammatory response can promote the production of ROS, while ROS can promote the production of inflammatory cytokines by activating NF‐*κ*B and initiating the NLRP3 inflammasome (Mittal et al. 2014). Alcohol can cause LSP and other pathogenic factors to enter the portal circulation and activate TLR receptors (Mello et al. 2009). After TLR activation, it mediates signal transduction through MyD88, then activates the nuclear factor NF‐*κ*B, initiates the NF‐*κ*B signaling pathway, promotes the release of the downstream proinflammatory factors IL‐1*β*, IL‐6, and TNF‐*α*, reduces the release of the anti‐inflammatory factor IL‐10, and finally leads to liver inflammation (Su et al. 2018). In this study, LPS in the MC group bound to TLRs and activated the MyD88 protein, which led to the activation of the NF‐*κ*B transcription factor. Activated NF‐*κ*B enters the nucleus to promote the secretion of IL‐1*β*, IL‐6, and TNF‐*α*. Compared to the free AST group, the ASTFAV group presented more significant inhibitory effects on the protein expression of TLRs, MyD88, and the proinflammatory factors IL‐1β, IL‐6, and TNF‐α, while the release of the anti‐inflammatory factor IL‐10 increased significantly (*p* < 0.05). These results confirmed that the hepatoprotective mechanism of AST‐FAV in ALD is realized through the TLR/MyD88/NF‐*κ*B signaling pathway.

Interleukin 10 (IL‐10) can control alcohol‐induced liver injury by inhibiting the proinflammatory cytokine TNF‐*α*, thus reducing alcohol‐induced ALD (Gao 2012). TNF‐α activates the nuclear factor NF‐*κ*B signaling pathway by binding to TNFR on the cell surface, which leads to apoptosis, leukocyte migration, and the secretion of proinflammatory factors, such as IL‐1*β*, IL‐6, and TNF‐*α* (Chen, Wolchok, and Bass 2021). In this study, AST significantly decreased the protein levels of TNF‐*α* and TNFR in ALD mice, and compared to free AST, AST‐FAV further enhanced this effect at the same dose. These results showed that AST‐FAV can improve alcohol‐induced ALD through the TNF‐*α*/TNFR/NF‐*κ*B pathway. This finding was similar to the results of another study (Hua et al. 2024).

In the inflammatory pathway, NLRP3 is a common initiator. The NLRP3 inflammasome activates the NF‐*κ*B signaling pathway, promoting the transcription and translation of NLRP3 in the nucleus (Liang et al. 2020), thus increasing the release of the inflammatory factors IL‐1*β* and IL‐18 (Feng et al. 2018), further causing inflammatory reactions. In this process, the NLRP3 inflammasome aggravates the inflammatory response of the body through the cell pyroptosis pathway, which depends on caspase‐1 (Muhammad et al. 2021). In this study, compared to that in the free AST group, the fluorescence intensity of NLRP3 was significantly lower after AST‐FAV intervention, thus reducing the fluorescence intensity of its downstream factor, caspase‐1, and the release of the inflammatory factors IL‐1*β* and IL‐18. The results of this study indicated that AST‐FAV can alleviate liver inflammation and pyroptosis caused by alcohol consumption and reduce liver damage in mice through the NLRP3/NF‐*κ*B pathway.

The conclusions of this study are primarily based on a mouse model. Although the mouse model is a commonly used and effective system for investigating the inhibitory mechanisms of astaxanthin on ALD, inherent differences in physiological metabolism and disease progression between mice and humans still exist, and the long‐term safety of AST‐FAV requires further evaluation. Nevertheless, the current data preliminarily confirm the safety and efficacy of AST‐FAV under the experimental conditions employed, providing a solid theoretical foundation and preliminary data support for future research. Subsequent studies will build upon these findings to further optimize the structure and function of AST‐FAV, conduct preclinical validation in higher animal models, and progressively advance its translation toward clinical research.

## 5 Conclusion

In conclusion, this study demonstrates that AST‐FAV more effectively interferes with abnormal lipid metabolism and oxidative stress‐induced liver injury than free AST and protects the liver from alcohol‐induced inflammation by regulating inflammatory factors and proteins through the TLR/MyD88/NF‐κB, TNF‐α/TNFR/NF‐κB, and NLRP3/NF‐κB inflammatory pathways. These findings highlight the ability of AST‐FAV to reduce ALD. Although AST holds therapeutic potential for ALD, its practical application is severely limited by its low bioavailability and poor stability. Encapsulating AST within fatty acid vesicles can significantly ameliorate these issues and greatly enhance its bioavailability. Furthermore, as a natural bioactive compound, astaxanthin not only exerts therapeutic effects but also effectively avoids the potential toxic side effects often associated with conventional pharmaceuticals, thereby offering a distinct advantage in terms of safety. However, considering the complexity and diversity of liver function, this mechanism is only one of many pathways involved. Future studies should investigate the mechanism underlying the protective effect of AST‐FAV on hepatocyte apoptosis induced by excessive alcohol consumption and its relationship with the intestinal microbiota.

## Author Contributions

**Chenchen Li:** project administration, data curation, conceptualization. **Xintong Zhang:** writing – original draft, methodology, data curation. **Chunran Han:** investigation. **Weiye Xiu:** data curation. **Diaomei Ma:** writing – review and editing, validation.

## Ethics Statement

All animal experimental procedures followed animal ethics guidelines and were approved by the Animal Ethics Committee of Harbin University of Commerce (Approval No. HSDU2024065, Harbin, China).

## Conflicts of Interest

The authors declare no conflicts of interest.

## Supporting information


**Figure S1:** Freeze‐dried powder (A); freeze‐drying and reconstitution (B); transmission electron microscope images of FAV, FAV‐AST (C); FTIR spectra of FAV, FAV‐AST, and AST (D).

## Data Availability

The original contributions presented in the study are included in the article; further inquiries can be directed to the corresponding author.

## References

[fsn372076-bib-0001] Boonlao, N. , S. Shrestha , M. B. Sadiq , and A. K. Anal . 2020. “Influence of Whey Protein‐Xanthan Gum Stabilized Emulsion on Stability and In Vitro Digestibility of Encapsulated Astaxanthin.” Journal of Food Engineering 272: 109859. 10.1016/j.jfoodeng.2019.109859.

[fsn372076-bib-0002] Braillon, A. , and F. Naudet . 2022. “Alcohol‐Related Liver Disease Treatment and EBM.” Liver International 42, no. 8: 1909. 10.1111/liv.15336. 35686691

[fsn372076-bib-0003] Buko, V. , I. Zavodnik , G. Budryn , et al. 2021. “Chlorogenic Acid Protects Against Advanced Alcoholic Steatohepatitis in Rats via Modulation of Redox Homeostasis, Inflammation, and Lipogenesis.” Nutrients 13, no. 11: 4155. 10.3390/nu13114155. 34836410 PMC8617701

[fsn372076-bib-0004] Chen, A. Y. , J. D. Wolchok , and A. R. Bass . 2021. “TNF in the Era of Immune Checkpoint Inhibitors: Friend or Foe?” Nature Reviews Rheumatology 17, no. 4: 213–223. 10.1038/s41584-021-00584-4. 33686279 PMC8366509

[fsn372076-bib-0005] Chen, Y. , Z. Jiang , J. Xu , et al. 2021. “Improving the Ameliorative Effects of Berberine and Curcumin Combination via Dextran‐Coated Bilosomes on Non‐Alcohol Fatty Liver Disease in Mice.” Journal of Nanobiotechnology 19, no. 1: 230. 10.1186/s12951-021-00979-1. 34348707 PMC8336351

[fsn372076-bib-0006] Clugston, R. D. 2020. “Carotenoids and Fatty Liver Disease: Current Knowledge and Research Gaps.” Biochimica et Biophysica Acta 1865, no. 11: 158597. 10.1016/j.bbalip.2019.158597. 31904420

[fsn372076-bib-0007] Feng, Y. , Y. Wang , P. Wang , Y. Huang , and F. Wang . 2018. “Short‐Chain Fatty Acids Manifest Stimulative and Protective Effects on Intestinal Barrier Function Through the Inhibition of NLRP3 Inflammasome and Autophagy.” Cellular Physiology and Biochemistry 49, no. 1: 190–205. 10.1159/000492853. 30138914

[fsn372076-bib-0008] Gao, B. 2012. “Hepatoprotective and Anti‐Inflammatory Cytokines in Alcoholic Liver Disease.” Journal of Gastroenterology and Hepatology 27, no. s2: 89–93. 10.1111/j.1440-1746.2011.07003.x. 22320924 PMC3281557

[fsn372076-bib-0009] Gao, B. , M. F. Ahmad , L. E. Nagy , and H. Tsukamoto . 2019. “Inflammatory Pathways in Alcoholic Steatohepatitis.” Journal of Hepatology 70, no. 2: 249–259. 10.1016/j.jhep.2018.10.023. 30658726 PMC6361545

[fsn372076-bib-0010] Gao, Y. , S. Yuan , Y. Chen , et al. 2022. “The Improvement Effect of Astaxanthin‐Loaded Emulsions on Obesity Is Better Than That of Astaxanthin in the Oil Phase.” Food & Function 13, no. 6: 3720–3731. 10.1039/D1FO03185F. 35266464

[fsn372076-bib-0011] Han, J. H. , J. H. Ju , Y. S. Lee , et al. 2018. “Responses via Blocking of STAT3.” Scientific Reports 8: 14090. 30237578 10.1038/s41598-018-32497-w PMC6148091

[fsn372076-bib-0012] Hua, Z. , X. Zhang , S. Xing , et al. 2024. “Design and Preparation of Multifunctional Astaxanthin Nanoparticles With Good Acid Stability and Hepatocyte‐Targeting Ability for Alcoholic Liver Injury Alleviation.” Materials Today Nano 25: 100436. 10.1016/j.mtnano.2023.100436.

[fsn372076-bib-0013] Jang, Y. J. , B. S. Cha , D. Kim , et al. 2023. “Extracellular Vesicles, as Drug‐Delivery Vehicles, Improve the Biological Activities of Astaxanthin.” Antioxidants 12, no. 2: 473. 10.3390/antiox12020473. 36830031 PMC9952194

[fsn372076-bib-0014] Jia, Y. , C. Wu , J. Kim , B. Kim , and S.‐J. Lee . 2016. “Astaxanthin Reduces Hepatic Lipid Accumulations in High‐Fat‐Fed C57BL/6J Mice via Activation of Peroxisome Proliferator‐Activated Receptor (PPAR) Alpha and Inhibition of PPAR Gamma and Akt.” Journal of Nutritional Biochemistry 28: 9–18. 26878778 10.1016/j.jnutbio.2015.09.015

[fsn372076-bib-0015] Kaur, M. , L. Kaur , G. Singh , L. Singh , A. Kaur , and R. K. Dhawan . 2023. “Recent Advancements in Biomimetic Drug Delivery System of Single‐Chain Fatty Acids as Ufasomes and Ufosomes: A Comprehensive Review.” Current Nanoscience 19, no. 3: 362–371. 10.2174/1573413718666220919113148.

[fsn372076-bib-0016] Kim, B. , C. Farruggia , C. S. Ku , et al. 2017. “Astaxanthin Inhibits Inflammation and Fibrosis in the Liver and Adipose Tissue of Mouse Models of Diet‐Induced Obesity and Nonalcoholic Steatohepatitis.” Journal of Nutritional Biochemistry 43: 27–35. 28193580 10.1016/j.jnutbio.2016.01.006

[fsn372076-bib-0017] Kim, M.‐S. 2016. “Optimal Management for Alcoholic Liver Disease: Conventional Medications, Natural Therapy or Combination?” World Journal of Gastroenterology 22, no. 1: 8. 10.3748/wjg.v22.i1.8. 26755857 PMC4698510

[fsn372076-bib-0018] Krestinina, O. , Y. Baburina , R. Krestinin , I. Odinokova , I. Fadeeva , and L. Sotnikova . 2020. “Astaxanthin Prevents Mitochondrial Impairment Induced by Isoproterenol in Isolated Rat Heart Mitochondria.” 10.3390/antiox9030262 PMC7139515 32210012

[fsn372076-bib-0019] Li, B. , Q. Mao , D. Zhou , et al. 2021. “Effects of Tea Against Alcoholic Fatty Liver Disease by Modulating Gut Microbiota in Chronic Alcohol‐Exposed Mice.” Food 10, no. 6: 1232. 10.3390/foods10061232. PMC8228948 34071491

[fsn372076-bib-0020] Liang, J. , Y. Liu , J. Liu , et al. 2018. “Chitosan‐Functionalized Lipid‐Polymer Hybrid Nanoparticles for Oral Delivery of Silymarin and Enhanced Lipid‐Lowering Effect in NAFLD.” Journal of Nanobiotechnology 16, no. 1: 64. 10.1186/s12951-018-0391-9. 30176941 PMC6122632

[fsn372076-bib-0021] Liang, Q. , W. Cai , Y. Zhao , et al. 2020. “Lycorine Ameliorates Bleomycin‐Induced Pulmonary Fibrosis via Inhibiting NLRP3 Inflammasome Activation and Pyroptosis.” Pharmacological Research 158: 104884. 10.1016/j.phrs.2020.104884. 32428667

[fsn372076-bib-0022] Liu, H. , X. Zhang , J. Xiao , et al. 2020. “Mitochondrial Dysfunction, and Regulating Metabolic Markers.” Cite This: Food & Function 11: 4103. 10.1039/d0fo00633e 32343758

[fsn372076-bib-0023] Lv, Y. , S. Hao , Y. Wang , S. Xing , and M. Tan . 2025. “Hepatocytes and Mitochondria Dual‐Targeted Astaxanthin WPI‐SCP Nanoparticles for the Alleviation of Alcoholic Liver Injury.” International Journal of Biological Macromolecules 285: 137992. 10.1016/j.ijbiomac.2024.137992. 39581423

[fsn372076-bib-0024] Mello, T. , S. Polvani , and A. Galli . 2009. “Peroxisome Proliferator‐Activated Receptor and Retinoic X Receptor in Alcoholic Liver Disease.” PPAR Research 2009, no. 1: 748174. 10.1155/2009/748174. 19756185 PMC2743826

[fsn372076-bib-0025] Mitra, S. , A. De , and A. Chowdhury . 2020. “Epidemiology of Non‐Alcoholic and Alcoholic Fatty Liver Diseases.” Translational Gastroenterology and Hepatology 5: 16. 10.21037/tgh.2019.09.08. 32258520 PMC7063528

[fsn372076-bib-0026] Mittal, M. , M. R. Siddiqui , K. Tran , S. P. Reddy , and A. B. Malik . 2014. “Reactive Oxygen Species in Inflammation and Tissue Injury.” Antioxidants & Redox Signaling 20, no. 7: 1126–1167. 10.1089/ars.2012.5149. 23991888 PMC3929010

[fsn372076-bib-0027] Muhammad, R. N. , L. A. Ahmed , R. M. Abdul Salam , K. A. Ahmed , and A. S. Attia . 2021. “Crosstalk Among NLRP3 Inflammasome, ETBR Signaling, and miRNAs in Stress‐Induced Depression‐Like Behavior: A Modulatory Role for SGLT2 Inhibitors.” Neurotherapeutics 18, no. 4: 2664–2681. 10.1007/s13311-021-01140-4. 34664178 PMC8804152

[fsn372076-bib-0028] Ni, Y. , M. Nagashimada , F. Zhuge , et al. 2015. “Astaxanthin Prevents and Reverses Diet‐Induced Insulin Resistance and Steatohepatitis in Mice: A Comparison With Vitamin E.” Scientific Reports 5, no. 1: 17192. 10.1038/srep17192. 26603489 PMC4658633

[fsn372076-bib-0029] Nowak, M. , A. S. Skwarecki , J. Pilch , et al. 2023. “Fatty Acids as Molecular Carriers in Cleavable Antifungal Conjugates.” European Journal of Medicinal Chemistry 252: 115293. 10.1016/j.ejmech.2023.115293. 36958265

[fsn372076-bib-0030] Penislusshiyan, S. 2020. “Novel Antioxidant Astaxanthin‐s‐Allyl Cysteine Biconjugate Diminished Oxidative Stress and Mitochondrial Dysfunction to Triumph Diabetes in Rat Model.” Life Sciences 245: 117367. 32001265 10.1016/j.lfs.2020.117367

[fsn372076-bib-0031] Salama, A. H. , and M. H. Aburahma . 2015. “Ufasomes Nano‐Vesicles‐Based Lyophilized Platforms for Intranasal Delivery of Cinnarizine: Preparation, Optimization, Ex‐Vivo Histopathological Safety Assessment and Mucosal Confocal Imaging.” Pharmaceutical Development and Technology 21, no. 6: 706–715. 10.3109/10837450.2015.1048553. 25996631

[fsn372076-bib-0032] Sandmann, G. 2025. “Origin and Evolution of Yeast Carotenoid Pathways.” Biochimica et Biophysica Acta, Molecular and Cell Biology of Lipids 1870, no. 2: 159586. 10.1016/j.bbalip.2024.159586. 39667662

[fsn372076-bib-0033] Sharma, K. , D. Sharma , M. Sharma , et al. 2018. “Astaxanthin Ameliorates Behavioral and Biochemical Alterations in In‐Vitro and In‐Vivo Model of Neuropathic Pain.” Neuroscience Letters 674: 162–170. 10.1016/j.neulet.2018.03.030. 29559419

[fsn372076-bib-0034] Shen, M. , K. Chen , J. Lu , et al. 2014. “Protective Effect of Astaxanthin on Liver Fibrosis Through Modulation of TGF‐ β 1 Expression and Autophagy.” Mediators of Inflammation 2014: 1–14. 10.1155/2014/954502. PMC4016904 24860243

[fsn372076-bib-0035] Su, Q. , X. Lv , Y. Sun , Z. Ye , B. Kong , and Z. Qin . 2018. “Role of TLR4/MyD88/NF‐κB Signaling Pathway in Coronary Microembolization‐Induced Myocardial Injury Prevented and Treated With Nicorandil.” Biomedicine & Pharmacotherapy 106: 776–784. 10.1016/j.biopha.2018.07.014. 29990871

[fsn372076-bib-0036] Sun, Y. , H. Shen , C. Fan , et al. 2024. “Typical Structural Characteristics and Hepatoprotective Effects of Novel High Fischer Ratio Oligopeptides From Antarctic Krill on Acute Alcoholic Liver Injury.” Food & Function 15, no. 18: 9298–9314. 10.1039/D4FO02609H. 39163024

[fsn372076-bib-0037] Wang, J. , X. Wang , W. Xiu , et al. 2024. “The Sweet Corn Cob Selenium Polysaccharide Alleviates Type 2 Diabetes via Modulation of LPS/IκBα/NFκB and the Intestinal Microbiota.” Food Bioscience 58: 103742. 10.1016/j.fbio.2024.103742.

[fsn372076-bib-0038] Wang, P. , X. Zheng , R. Du , et al. 2023. “Astaxanthin Protects Against Alcoholic Liver Injury via Regulating Mitochondrial Redox Balance and Calcium Homeostasis.” Journal of Agricultural and Food Chemistry 71, no. 49: 19531–19550. 10.1021/acs.jafc.3c05529. 38038704

[fsn372076-bib-0039] Wang, Q. , Y. Zhao , L. Guan , et al. 2017. “Preparation of Astaxanthin‐Loaded DNA/Chitosan Nanoparticles for Improved Cellular Uptake and Antioxidation Capability.” Food Chemistry 227: 9–15. 10.1016/j.foodchem.2017.01.081. 28274463

[fsn372076-bib-0040] Wu, Y. C. , H. H. Huang , Y. J. Wu , I. Manousakas , C. C. Yang , and S. M. Kuo . 2019. “Therapeutic and Protective Effects of Liposomal Encapsulation of Astaxanthin in Mice With Alcoholic Liver Fibrosis.” International Journal of Molecular Sciences 20, no. 16: 4057. 10.3390/ijms20164057. 31434227 PMC6718996

[fsn372076-bib-0041] Yadav, R. , N. Adikessavane , R. R. Mahato , and S. Maiti . 2025. “Decoding Information Entropy of Fatty Acid and Phospholipid Vesicles via Ordering Combinatorial Output of Hydrazones.” Chemical Science 16, no. 37: 17184–17192. 10.1039/D5SC04365D. 40904484 PMC12402723

[fsn372076-bib-0042] Yaghooti, H. , N. Mohammadtaghvaei , and K. Mahboobnia . 2019. “Effects of Palmitate and Astaxanthin on Cell Viability and Proinflammatory Characteristics of Mesenchymal Stem Cells.” International Immunopharmacology 68: 164–170. 10.1016/j.intimp.2018.12.063. 30639962

[fsn372076-bib-0043] Yang, C. , Y. I. Hassan , R. Liu , et al. 2019. “Anti‐Inflammatory Effects of Different Astaxanthin Isomers and the Roles of Lipid Transporters in the Cellular Transport of Astaxanthin Isomers in Caco‐2 Cell Monolayers.” Journal of Agricultural and Food Chemistry 67, no. 22: 6222–6231. 10.1021/acs.jafc.9b02102. 31117505

[fsn372076-bib-0044] Yang, X. , B. Wang , H. Zeng , et al. 2025. “A Modified Polydopamine Nanoparticle Loaded With Melatonin for Synergistic ROS Scavenging and Anti‐Inflammatory Effects in the Treatment of Dry Eye Disease.” Advanced Healthcare Materials 14: 2404372. 10.1002/adhm.202404372. 39828670

[fsn372076-bib-0045] Zahariev, N. , P. Katsarov , P. Lukova , and B. Pilicheva . 2023. “Novel Fucoidan Pharmaceutical Formulations and Their Potential Application in Oncology—A Review.” Polymers 15, no. 15: 3242. 10.3390/polym15153242. 37571136 PMC10421178

[fsn372076-bib-0046] Zakir, F. , B. Vaidya , A. K. Goyal , B. Malik , and S. P. Vyas . 2010. “Development and Characterization of Oleic Acid Vesicles for the Topical Delivery of Fluconazole.” Drug Delivery 17, no. 4: 238–248. 10.3109/10717541003680981. 20235758

[fsn372076-bib-0047] Zhao, L. , L. Li , Y. Zhang , et al. 2024. “Targeting Synovial Macrophages With Astaxanthin‐Loaded Liposomes for Antioxidant Treatment of Osteoarthritis.” ACS Biomaterials Science & Engineering 10, no. 11: 7191–7205. 10.1021/acsbiomaterials.4c00998. 39413302

[fsn372076-bib-0048] Zhou, Z.‐S. , C.‐F. Kong , J.‐R. Sun , X.‐K. Qu , J.‐H. Sun , and A.‐T. Sun . 2022. “Fisetin Ameliorates Alcohol‐Induced Liver Injury Through Regulating SIRT1 and SphK1 Pathway.” American Journal of Chinese Medicine 50, no. 8: 2171–2184. 10.1142/S0192415X22500938. 36266756

[fsn372076-bib-0049] Zuo, A. , S. Wang , L. Liu , Y. Yao , and J. Guo . 2019. “Understanding the Effect of Anthocyanin Extracted From *Lonicera caerulea* L. on Alcoholic Hepatosteatosis.” Biomedicine & Pharmacotherapy 117: 109087. 10.1016/j.biopha.2019.109087. 31195351

