# Supplementary material for: Astaxanthin Vesicles Improve Alcoholic Liver Disease Through Oxidative Stress and NF‐κB Inflammatory Pathway
Source: Food Sci Nutr. 2026 Jul 1;14(7):e72076. doi: 10.1002/fsn3.72076 (PMC13322658; doi:10.1002/fsn3.72076)
Supplement: Supplementary file 1 — Figure S1: Freeze‐dried powder (A); freeze‐drying and reconstitution (B); transmission electron microscope images of FAV, FAV‐AST (C); FTIR spectra of FAV, FAV‐AST, and AST (D). [file FSN3-14-e72076-s001.docx]

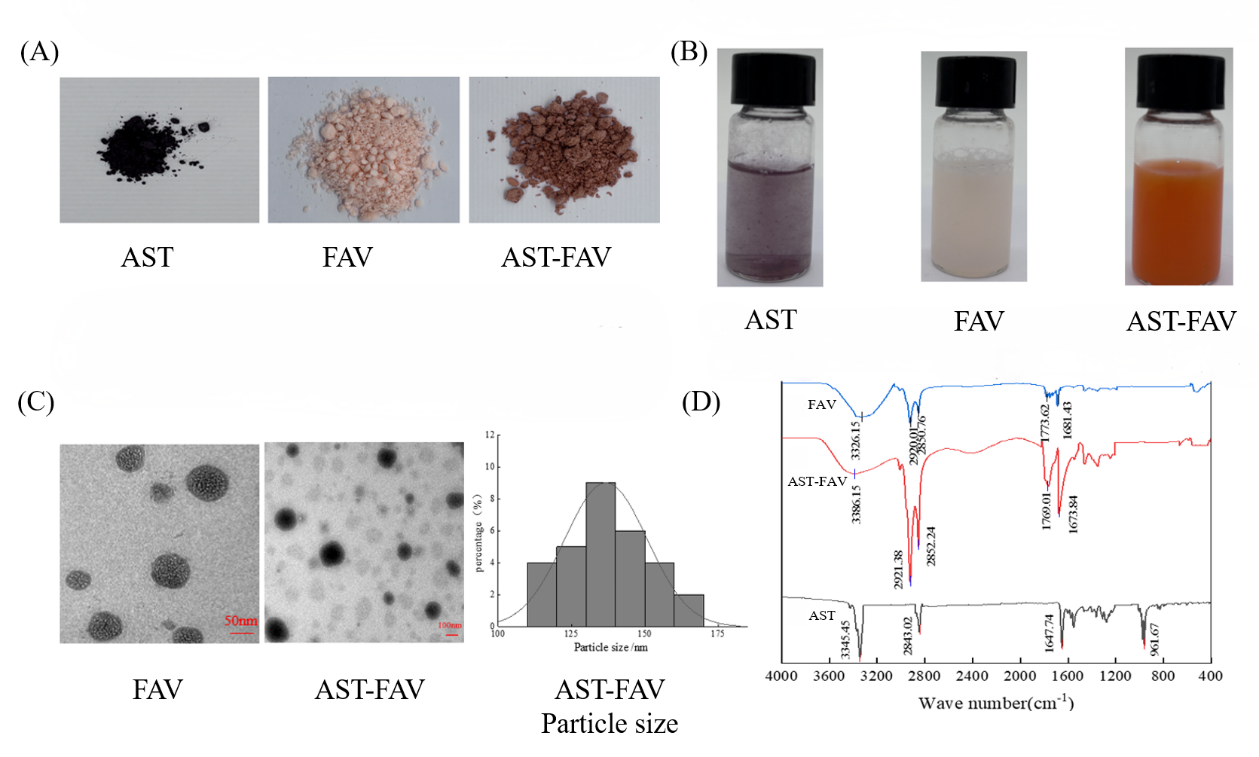


Figure. S1. Freeze-dried powder (A); Freeze-drying and reconstitution (B); Transmission electron microscope images of FAV, FAV-AST (C); FTIR spectra of FAV, FAV-AST, and AST (D).
